# Reduced expressions of connexin 43 and VEGF in the first-trimester tissues from women with recurrent pregnancy loss

**DOI:** 10.1186/s12958-016-0179-4

**Published:** 2016-08-17

**Authors:** Xiaoping He, Qinfang Chen

**Affiliations:** Department of Family Planning, The International Peace Maternity and Child Health Hospital, School of Medicine, Shanghai Jiao Tong University, Shanghai, 200030 China

**Keywords:** Recurrent pregnancy loss, Connexin 43, Vascular endothelial growth factor

## Abstract

**Background:**

Approximately 45–50 % of the recurrent pregnancy loss (RPL) remain(s) unexplained that challenges its clinical management. Formation and development of placenta as well as angiogenesis are critical for successful pregnancy. Vascular endothelial growth factor (VEGF) and connexin 43 (Cx43) play important roles in angiogenesis and placenta development and aberration of these have been linked to RPL. We aimed to investigate whether the expressions of VEGF and Cx43 were altered in the first-trimester tissues (chorionic villi and decidua) collected from women with RPL compared to those from healthy early pregnant women.

**Methods:**

First-trimester chorionic villi and decidua were collected from pregnant women diagnosed RPL who ended up with surgical intervention (*n* = 28) in comparison to those collected from women requesting surgical termination of their unwanted normal first-trimester pregnancies (*n* = 28). These two groups of women were matched in age and gestational weeks. Tissues were analyzed for the protein and messenger ribonucleic acid (mRNA) expressions of Cx43 and VEGF by immunohistochemistry, western blot, and quantitative reverse transcription polymerase chain reaction (qRT-PCR).

**Results:**

The expressions of both Cx43 and VEGF at the level of mRNA and protein in the villi and decidua from women with RPL were significantly decreased compared with those from women with normal early pregnancy.

**Conclusions:**

Reduction of Cx43 and VEGF expressed in the first-trimester tissues might indicate their important roles involved in RPL and thus holds the potential to develop pharmaceutical therapies for treatment of RPL.

**Electronic supplementary material:**

The online version of this article (doi:10.1186/s12958-016-0179-4) contains supplementary material, which is available to authorized users.

## Background

Recurrent pregnancy loss (RPL) refers to two or more consecutive pregnancy losses before 20 weeks of gestation defined by the American Congress of Obstetricians and Gynecologists (ACOG) [1, 2], and three or more pregnancy losses defined by Royal College of Obstetricians and Gynaecologists (RCOG) [3]. In China, for women with two early pregnancy losses, clinical evaluation is often recommended, and that conforms to the opinion of ACOG committee [4]. Recurrent pregnancy loss occurs about in 1 out of 100 pregnancies [5], causing depression, anxiety, stress and lowered self-esteem in couples suffering from RPL [6, 7]. However, the causes for approximately 45–50 % of the RPL patients remain unclear and that challenges clinical management of RPL [8].

During embryonic development, vascular endothelial growth factor (VEGF), a signal cytokine that stimulates vasculogenesis and angiogenesis [9], functions to create new blood vessels [10–12]. These suggest that impairment of angiogenesis and embryo development in RPL may be related to VEGF dysregulation. However, the direct relationship between VEGF and RPL remains debatable. While some studies linked the polymorphisms of VEGF gene with increased risk of RPL and decreased expression of VEGF gene in endometrium was found in pregnant women with unexplained RPL [13, 14]. Others found increased expressions of VEGF and VEGF receptor-1 (VEGFR-1) in serum and chorionic villi were associated with early RPL [15, 16]. In this study, we aimed to find out if VEGF expression in the first-trimester tissues was altered in women with RPL versus those with normal early pregnancy.

Connexin 43 (Cx43), one major component of cell-cell gap junction facilitating small soluble molecules exchange [17], is involved in embryo implantation and placenta formation. Reduced Cx43 expression may be correlated with RPL [17, 18]. Reduced Cx43 expression caused decreased expression of VEGF in endothelial progenitor cells, aortic endothelial cells, and subsequent impaired angiogenic potential [19–21]. Thus, we hypothesized that the expression(s) of Cx43 and/or VEGF in chorionic villi or decidua were reduced in women with RPL compared to that expressed in normal first-trimester tissues.

## Methods

The study was approved by the Ethical Committee of Medical Research at the International Peace Maternity and Child Health Hospital, Shanghai, China. Written informed consent forms were obtained from all subjects.

### Sample collection

Between October 2012 and September 2013, 28 women diagnosed with embryo death by ultrasound during the first 12 weeks of pregnancy who had already experienced two or more miscarriages were recruited. All of them were diagnosed unexplained RPL and received surgical intervention upon diagnosis. During surgery, the decidua and chorionic villi were collected from them (*n* = 28) as well as from women with normal early pregnancy who requested surgical abortion for their unwanted pregnancies serving as a control group (*n* = 28) at the same gestational age. Women classified to the control group had the history of prior healthy live births and had no abnormal pregnancy history including previous miscarriage, ectopic pregnancy, and still birth. The two groups of women were matched in age and gestational age. From each woman, half of the samples (decidua and chorionic villi) were fixed in 10 % formalin, dehydrated in upgraded ethanol, and embedded in paraffin for immunostaining analysis and the other half of the samples were snap frozen in liquid nitrogen and stored at −80 °C for further analyses.

### Immunohistochemistry

The 5 μm-thickness paraffin sections were deparaffinized followed by rehydration in upgrading ethanol. Endogenous peroxidase activity was quenched using 0.3 % hydrogen peroxide in tris buffered saline(TBS) for 30 min. Epitope retrieval was performed in 0.1 M citrate buffer (pH 6.0) using a microwave at full power for 10 min. Sections were incubated with appropriate blocking serum (VECTASTAIN UNIVERSAL ABC kit, VECTOR) at room temperature for 20 min followed by incubation with diluted mouse anti human Cx43 (Cell Signaling Technology) or rabbit anti human VEGF (Abcam) overnight in a humidified chamber at 4 °C. Negative controls were included by replacing the primary antibody with TBS. After rinsing in TBS three times with 5 min each, sections with incubated with appropriate secondary antibody for 30 min at room temperature. Following rinse, diamino-benzidine substrate (DAB kit, VECTOR, CA, USA) was applied to the sections for 4 min. Slides were cover-slipped and five views per section were taken under 400X magnification. Blinded sample coding was applied and the staining was independently evaluated by two pathologists using a semi-quantitative immuno-reactivity score. The staining for each sample was scored by multiplying the percentage of positive staining (PP value: 0: zero, 1: < 10 %, 2: 10–50 %, 3: > 50 %) with the intensity of staining (SI value: 0: negative, 1: weak, 2: moderate, 3: strong) [22]. The final results were generated by averaging the scores over the five views.

### Western blot

About one cubic meter of the decidua and villi tissues were minced, mixed with protease inhibitor cocktail (Thermo Scientific), homogenized, and centrifuged at 12000 rpm in 4 °C. The concentration of total protein was determined using a BCA assay (Sigma, CA, USA) according to the manufacturer’s instruction. Forty micrograms of protein were loaded onto gel lanes, separated by sodium dodecyl sulfate polyacrylamide gel electrophoresis (SDS-PAGE), and then transferred onto polyvinylidene fluoride (PVDF) membranes (Millipore, Bedford, MA). After incubation in blocking buffer (Beyotime, Shanghai) at room temperature for two hours, the membranes were then incubated overnight with the primary antibody of Cx43 or VEGF at 4 °C. After rinsing in TBS three times for 5 min each, the membranes were incubated with horseradish peroxidase (HRP)-conjugated secondary antibody (Abcam) for two hours at room temperature. The signal was visualized using enhanced chemiluminescence western blotting detection reagents (Pierce) on FluorChem E System Instrument. The band intensity was analyzed using Alphaview software.

### qRT-PCR

About one cubic meter of the decidua and villi were homogenized. Total RNA was isolated using TRIZOL reagent (Invitrogen, USA) according to the manufacturer’s instruction. The PrimeScriptTM RT reagent kit with genomic deoxyribonucleic acid (gDNA) Eraser (Takara, Japan) was used to remove the genomic DNA and synthesized complementary DNA (cDNA). The PCR amplification was performed on MasterCycler RealPlex 4 (Eppendorf, USA) using SYBRTM Premix Ex TaqTM II Kit (Takara) according to the manufacturer’s instructions. The reaction mixture in a 20 μL volume contained 2 μL (100 ng) of cDNA, 1.6 μL (0.4 μM) mixture of the forward and reverse primers (sequences listed in Table 1), 10 μL of SYBR Premix Ex Taq II (2X) and 6.4 μL of RNase-free water. The reaction conditions were two minutes at 95 °C of initial denaturation, 40 cycles of denaturation for seven seconds at 95 °C, and annealing/extension for 30 sections at 60 °C. The cycle threshold (Ct) was determined and fold changes of the interest genes relative to Glyceraldehyde 3-phosphate dehydrogenase (GAPDH) serving as a house-keeping gene were calculated by the formula of the 2^-ΔΔCt^. The qRT-PCR experiment was repeated three times with duplicates of each sample.

**Table 1:** Sequences of primers used for qRT-PCR analysis

Gene name	Forward (5′–3′)	Reverse (5′–3′)
Cx43	AGTTCAATCACTTGGCGTGACTT	GCAGTTGAGTAGGCTTGAACCTT
VEGF	TGCTGTCTTGGGTGCATT	GCATGGTGATGTTG
GAPDH	GGATTTGGTCGTATTGGG	CTGGAAGATGGTGATGGGATT

### Statistical analyses

The Statistical Package for the Social Sciences (SPSS, version 13.0) was used to perform all statistical analyses. Data were checked for normality and equal variance. Student’s t-test was used for parametric data, while Mann-Whitney test was applied for non-parametric data to compare the fold change of gene expression or immunostaining score between the RPL group and control groups. The association between the Cx43 and VEGF expressions was analyzed using linear correlation analysis. *P* < 0.05 was considered statistically significant. The values were presented as mean ± Standard Deviation (SD).

## Results

There was no difference in age (30.3 ± 4.0 versus 28.7 ± 5.1) or gestational days (62.8 ± 8.3 versus 59.1 ± 7.6) between the RPL and control groups.

### Protein and mRNA expressions of VEGF in RPL group versus control group

**Fig. 1:**
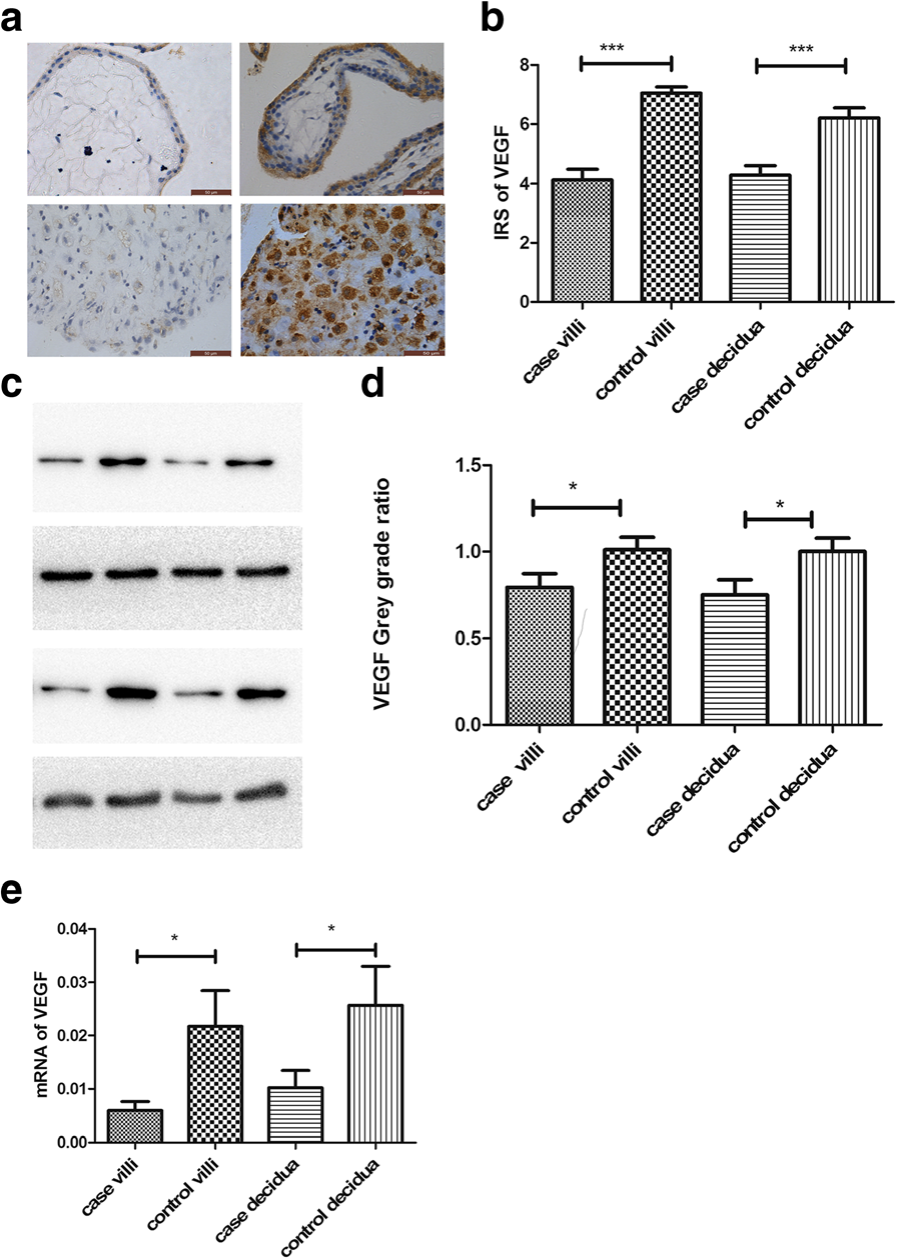
The expression of VEGF in chorionic villi and decidua collected from RPL women and control group. **a**, **b** The protein expression of VEGF was assessed by immunohistochemistry (magnification X400) and evaluated by IRS scores. The control group exhibited a higher level of VEGF than RPL group. **c**, **d** The VEGF protein expression was assessed by Western Blot and the band intensity was evaluated. The VEGF protein is markedly more in the control group than that in the RPL group. **e** The mRNA level of VEGF was significantly lower in the PRL group than that in the control group. Data were presented as mean ± standard deviation (SD). *: *P* < 0.05; ***: *P* < 0.001

Compared to the control group, the immunoreactivity of VEGF in either chorionic villi or decidua was dramatically reduced in RPL group than that in control group as revealed by immunostaining (Fig. 1a, b). This is consistent with the results of western blot (Fig. 1c, d). Similarly, the level of VEGF mRNA was also significantly lower in the PRL group than that in the control group (Fig. 1e).

### The protein and mRNA expressions of Cx43 in RPL group versus control group

**Fig. 2:**
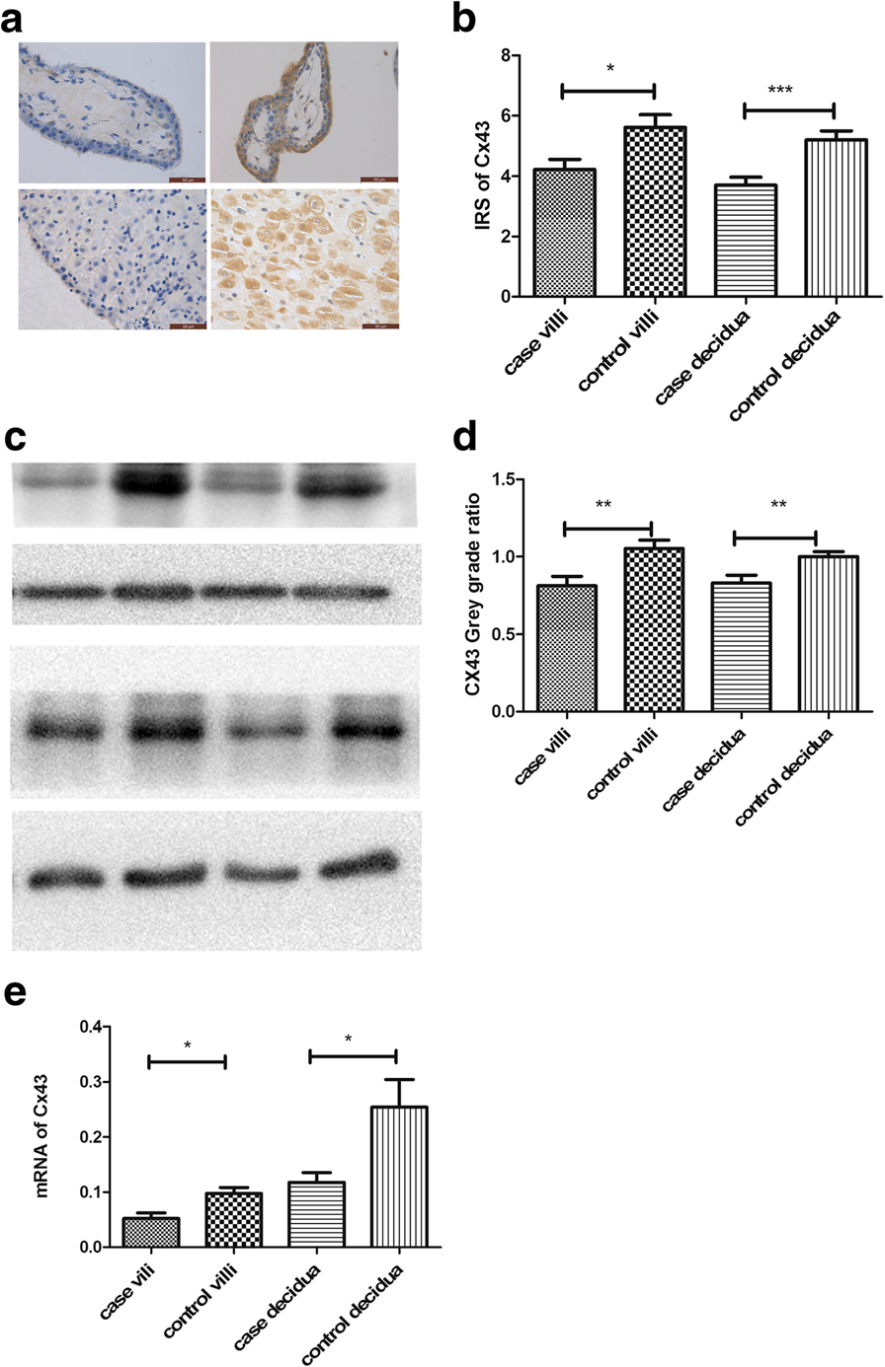
The expression of Cx43 in chorionic villi and decidua collected from RPL women and control group. **a**, **b** The protein expression of Cx43 was measured by immunohistochemistry (magnification X400) and evaluated by IRS scores. The control group exhibited a higher level of Cx43 protein compared to the RPL group. **c**, **d** The Cx43 protein expression was assessed by Western Blot and the band intensity was evaluated. The protein level of Cx43 is significantly higher in the control group than that in the RPL group. **e** The expression of Cx43 mRNA was reduced in both villi and decidua from the RPL group and those from the control group. Data were presented as mean ± standard deviation (SD). *: *P* < 0.05; **P < 0.01; ***: *P* < 0.001

We found that Cx43 was predominantly expressed in the cytoplasm of cytotrophoblast, syncytiotrophoblast, and decidua. Both immunohistochemistry (Fig. 2a, b) and western blot (Fig. 2c, d) showed the protein level of Cx43 expressed in either chorionic villi or decidua was significantly lower in the RPL group than that in controls. The expression of Cx43 mRNA showed the same tendency of reduction in the RPL group compared to that in the control group (Fig. 2e).

### Correlation between VEGF and Cx43 expression in chorionic villi and decidua

**Fig. 3:**
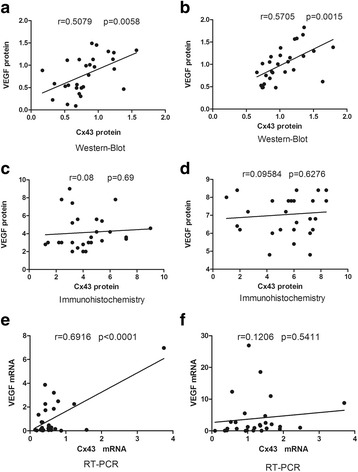
Relationship of Cx43 and VEGF expressed in the chorionic villi collected from RPL women versus controls. The protein levels of Cx43 and VEGF expressed in the chorionic villi were positively and linearly related as revealed by western blot in both RPL group (**a**) and control group (**b**), though the immunohistochemical result did not find any correlation in either group (c, **d**). The same positive and linear relationship was detected in the mRNA expression in the RPL group (**e**), but not in the control group (**f**)

**Fig. 4:**
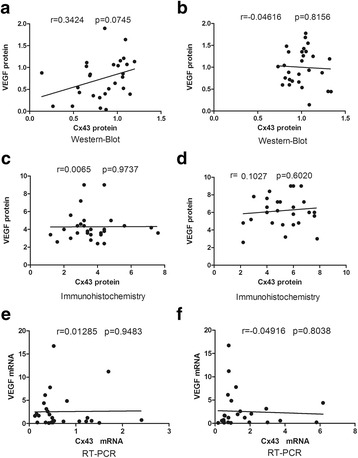
Relationship of Cx43 and VEGF expressed in the decidua collected from RPL women versus controls. In the decidua, there was no significant correlation of protein expression of Cx43 and VEGF from RPL women (**a**, **c**) or the control group (**b**, **d**). This is consistent with the results of mRNA expression in both groups (**e**, **f**)

Both protein and mRNA expressions of VEGF and Cx43 in the chorionic villi collected from women with RPL were positively and linearly related as revealed by western blot and qRT-PCR (Fig. 3c, e), though immunohistochemical result did not support that. In the control group, the result from western blot, but not from immunostaining, showed that the VEGF and Cx43 protein levels in the chorionic villi were significantly correlated in a positive manner. In the decidua, there was no significant correlation between Cx43 and VEGF expressions in either RPL group (Fig. 3b, d, f) or control group (Fig. 4b, d, f).

## Discussion

The primary biological function of VEGF is promoting microvessel permeability and angiogenesis. The development of embryo needs extensive and systematic blood vessels for supporting implantation and development, which is impaired in RPL [23, 24]. VEGF induces endothelial cell proliferation and migration while decreasing cell apoptosis, increases vascular permeability, and accelerates stromal proteolysis [25]. VEGF is a critical factor involved in the angiogenic development of fetus and placenta, and embryo implantation and development, and decidualization [26, 27]. Researchers have found increased expression of VEGF and its soluble Fms-like tyrosine kinase-1 (sFlt-1) in normal placenta development, suggesting that VEGF signaling is essential for vessel formation and angiogenesis in placenta and developing embryo. One of the pathological features of RPL is dysfunctional vessel formation and angiogenesis, implicating that VEGF dysregulation may be related to RPL. Genetic study has demonstrated that angiogenesis genes including VEGF function for maintaining normal pregnancy and is correlated with RPL [28]. Several VEGF-A gene single nucleotide polymorphisms (SNPs) associated with spontaneous abortion have also been identified [29]. Choi et al. found that the VEGF gene expression in chorionic villi was lower in RPL group [28]. Amirchaghmaghi et al. demonstrated that VEGF gene expression in endometrial samples was lower in the RPL women compared with that in proven fertile females. Consistent with these findings, our work demonstrated that both mRNA and protein levels of VEGF are reduced in RPL as compared with those in controls. The increase in uterine vascular resistance and decrease in uterine blood flow inhibited placental angiogenesis contributing to early embryonic mortality [27]. Considering the above, we speculate that reduced expression of VEGF in RPL patients contribute to poor vessel formation and may result in recurrent miscarriage.

Some studies demonstrated that reduced expression of Cx43 might lead to vessel dysfunction and angiogenesis impairment [19–21]. Deletion of Cx43 gene impaired decidualization and resulted in poor angiogenesis [30]. Another study demonstrated that Cx43 mutation impaired decidual angiogenesis in mice with reduced expression of Cx43, but increased level of VEGF [31–33]. We found decreased expression of both VEGF and Cx43 expression in RPL, indicating that Cx43 and VEGF may interact with each other to maintain normal placenta structure and function during pregnancy. Placenta formation is essential to the maintenance and progress of pregnancy. A key step in this process is the fusion of cytotrophoblasts with syncytiotrophoblasts. The syncytiotrophoblast layer on the villous surface directly invades the endometrium which called implantation, rupturing maternal capillaries and thus participating in maternal-fetal material exchange. An in vitro experiment demonstrated that upregulation of Cx43 expression promoted gap junctional intercellular communication during the differentiation of cytotrophoblasts into syncytiotrophoblasts [34]. Gerbaud et al. found that Cx43 accelerated trophoblast fusion process by activating protein kinase A (PKA)-dependent phosphorylation [35]. Cx43 is an important component for gap junction formation in human preimplantation embryos [36]. Rmanathal et al. found that Cx43 played critical role in controlling the paracrine secretions of stromal cells under decidualization, which may alter the proliferation and migration of endothelial cells. And that can have impact on angiogenesis [37]. Consistent to these findings [18], we found reduced expression of Cx43 in first-trimester tissues from RPL patients. We speculate that reduced expression of Cx43 may lead to abnormal embryo implantation and poor angiogenesis, which may be related to RPL occurrence.

Our correlation analyses indicate that reduced expressions of Cx43 and VEGF may individually or interactively play roles in the occurrence of RPL.

In this study, the expression of Cx43 and VEGF in additional group of patients who underwent their first unexplained missed abortions has not been analyzed. In the further study, this group can be employed to explore the relation between the expression of the Cx43 and VEGF and the times they suffered unexplained missed abortions.

## Conclusions

We found that the expressions of VEGF and Cx43 are significantly reduced in chorionic villi and decidua from RPL women. And the reduction of both expressions may contribute to RPL by interacting with each other to influence the angiogenesis of placenta and developing embryo. Our study holds the potential to develop preventive or therapeutic strategies for RPL by targeting the molecules of VEGF and Cx43.

## Abbreviations

ACOG, American Congress of Obstetricians and Gynecologists; cDNA, complementary DNA; Ct, cycle threshold; Cx43, connexin 43; gDNA, genomic deoxyribonucleic acid; HRP, horseradish peroxidase; mRNA, messenger ribonucleic acid; PKA, protein kinase A; PP value, percentage of positive staining; PVDF, polyvinylidene fluoride; qRT-PCR, quantitative reverse transcription polymerase chain reaction RT-PCR; RCOG, Royal College of Obstetricians and Gynecologists; RPL, recurrent pregnancy loss; SD, Standard Deviation; SDS-PAGE, sodium dodecyl sulfate polyacrylamide gel electrophoresis; sFlt-1, soluble Fms-like tyrosine kinase-1; SI value, intensity of staining; SNPs, single nucleotide polymorphisms; SPSS, Statistical Package for the Social Sciences; TBS, tris buffered saline; VEGF, Vascular endothelial growth factor; VEGFR-1, VEGF receptor-1

## Additional file

Additional file 1: Supplementary document. (DOC 182 kb)
