# Supplementary material for: Reduced expressions of connexin 43 and VEGF in the first-trimester tissues from women with recurrent pregnancy loss
Source: Reprod Biol Endocrinol. 2016 Aug 17;14:46. doi: 10.1186/s12958-016-0179-4 (PMC4989327; doi:10.1186/s12958-016-0179-4)
Supplement: Additional file 1: — Supplementary document. (DOC 182 kb) [file 12958_2016_179_MOESM1_ESM.doc]

Control group

| No. | Age | Gestational days | Immunohistochemistry  scores | | | | Western blot  band intensity | | | |
| --- | --- | --- | --- | --- | --- | --- | --- | --- | --- | --- |
|  |  |  | Cx43 | | VEGF | | Cx43 | | VEGF | |
|  |  |  | villi | decidua | villi | decidua | villi | decidua | villi | decidua |
| 1 | 26 | 52 | 5.6 | 2.2 | 7.8 | 2.6 | 0.74887 | 1.08915 | 0.4841 | 0.14318 |
| 2 | 36 | 66 | 4.4 | 4.8 | 6 | 7.2 | 1.00612 | 1.05183 | 1.20075 | 1.35992 |
| 3 | 31 | 60 | 5 | 4.8 | 6.2 | 6.2 | 1.50468 | 0.85333 | 1.29749 | 1.3514 |
| 4 | 29 | 64 | 7.4 | 3 | 7.8 | 7.8 | 0.85051 | 0.8141 | 0.68779 | 0.75517 |
| 5 | 26 | 55 | 7.6 | 6.4 | 6.8 | 4.8 | 0.65774 | 1.30388 | 0.82105 | 0.44793 |
| 6 | 30 | 51 | 7.2 | 5 | 4.8 | 7.2 | 1.63751 | 0.8634 | 0.61379 | 0.71861 |
| 7 | 30 | 60 | 5.8 | 6.6 | 7.8 | 9 | 1.79238 | 1.00426 | 1.3818 | 1.352 |
| 8 | 34 | 60 | 8.4 | 6 | 8.4 | 9 | 0.96903 | 0.80277 | 1.00606 | 1.0433 |
| 9 | 23 | 60 | 6 | 6 | 6.2 | 6.6 | 0.93783 | 1.07407 | 0.47879 | 1.5488 |
| 10 | 25 | 52 | 7.4 | 6.8 | 6.2 | 7.2 | 0.8675 | 1.04796 | 1.17078 | 1.77577 |
| 11 | 42 | 64 | 5.4 | 2.2 | 7.2 | 4.8 | 1.34416 | 0.73113 | 1.66263 | 1.01532 |
| 12 | 35 | 48 | 1.8 | 4 | 8.4 | 6 | 0.87842 | 1.02052 | 0.83789 | 1.26318 |
| 13 | 20 | 55 | 4.4 | 6 | 7.8 | 4.6 | 1.0773 | 1.00545 | 0.82854 | 0.53665 |
| 14 | 25 | 50 | 6 | 5.4 | 5.4 | 3.2 | 0.79252 | 1.33689 | 0.75332 | 0.44246 |
| 15 | 22 | 63 | 2.6 | 4 | 7.2 | 6 | 1.3521 | 0.82846 | 1.18337 | 1.48147 |
| 16 | 33 | 67 | 6.2 | 4 | 6.6 | 6.6 | 1.15078 | 0.78315 | 1.1493 | 0.5961 |
| 17 | 32 | 50 | 6 | 7.2 | 7.8 | 6 | 0.76604 | 0.85395 | 0.5588 | 0.88376 |
| 18 | 30 | 80 | 8.4 | 6 | 7.8 | 9 | 1.36016 | 0.86113 | 1.82717 | 0.59742 |
| 19 | 30 | 69 | 6 | 7.6 | 7.8 | 6 | 1.20473 | 0.97024 | 0.87799 | 0.84033 |
| 20 | 22 | 60 | 8.4 | 5.8 | 8.4 | 9 | 0.89156 | 1.08558 | 0.81551 | 0.93222 |
| 21 | 33 | 63 | 7.2 | 5.2 | 6.8 | 4.6 | 0.72049 | 1.32016 | 0.52724 | 1.1915 |
| 22 | 23 | 60 | 1 | 6 | 8 | 5.8 | 0.74034 | 1.00369 | 1.01757 | 1.14551 |
| 23 | 30 | 64 | 7.8 | 4 | 8.4 | 7.6 | 1.01007 | 1.13193 | 1.36926 | 1.24898 |
| 24 | 21 | 48 | 7.2 | 7.8 | 6 | 3 | 0.84667 | 0.89488 | 1.05252 | 0.68474 |
| 25 | 27 | 68 | 6.2 | 4.2 | 8.4 | 8.4 | 1.22057 | 1.044 | 1.56635 | 1.68038 |
| 26 | 27 | 58 | 2 | 2.8 | 6.2 | 5.2 | 1.12184 | 1.02647 | 1.05934 | 1.42649 |
| 27 | 32 | 60 | 4.2 | 4.4 | 4.8 | 4.8 | 1.25176 | 0.96989 | 1.58024 | 0.66268 |
| 28 | 29 | 48 | 1.8 | 7.6 | 6.4 | 5.6 | 0.73809 | 1.23574 | 0.48048 | 0.87478 |

Case group

| No. | Age | Gestational days | Immunohistochemistry  scores | | | | Western blot  band intensity | | | |
| --- | --- | --- | --- | --- | --- | --- | --- | --- | --- | --- |
|  |  |  | Cx43 | | VEGF | | Cx43 | | VEGF | |
|  |  |  | villi | decidua | villi | decidua | villi | decidua | villi | decidua |
| 1 | 23 | 53 | 4 | 2 | 2 | 4 | 1.15046 | 0.72627 | 1.30874 | 0.35619 |
| 2 | 34 | 75 | 1.4 | 4.4 | 3 | 3.8 | 0.87475 | 0.56784 | 1.01032 | 0.42841 |
| 3 | 26 | 64 | 2.4 | 3.4 | 7.8 | 3.8 | 0.34682 | 0.26506 | 0.58748 | 0.11357 |
| 4 | 31 | 68 | 4.6 | 7.6 | 3.6 | 3.6 | 0.71746 | 1.0705 | 1.12804 | 0.52468 |
| 5 | 27 | 55 | 5.4 | 3 | 5.4 | 7.2 | 0.50878 | 1.19681 | 0.52939 | 1.13581 |
| 6 | 31 | 55 | 3 | 3.2 | 9 | 4.4 | 0.55497 | 0.79942 | 0.55319 | 0.3109 |
| 7 | 30 | 56 | 2.4 | 4.4 | 3 | 2.4 | 0.32089 | 0.97447 | 0.22451 | 0.6416 |
| 8 | 31 | 59 | 3.2 | 3 | 2 | 4.6 | 0.54118 | 0.80728 | 0.12196 | 0.07164 |
| 9 | 23 | 68 | 9 | 4 | 4.6 | 2.4 | 0.85673 | 1.06099 | 0.78088 | 0.78232 |
| 10 | 32 | 58 | 6 | 5.6 | 4.2 | 4.4 | 0.71745 | 0.87242 | 0.36078 | 0.03821 |
| 11 | 34 | 72 | 3.6 | 4.4 | 2.4 | 4 | 0.68267 | 0.8572 | 0.56661 | 1.89539 |
| 12 | 31 | 69 | 5 | 3.6 | 5.6 | 3.4 | 0.16787 | 1.09416 | 0.88486 | 1.63929 |
| 13 | 28 | 47 | 4 | 4.8 | 2.8 | 5 | 0.73835 | 0.90297 | 0.30431 | 0.51336 |
| 14 | 33 | 66 | 4.6 | 2.4 | 3 | 5.6 | 1.23265 | 0.54995 | 1.2825 | 0.54243 |
| 15 | 29 | 54 | 3.6 | 7.2 | 5.6 | 4.2 | 1.56901 | 0.68642 | 1.33646 | 0.81545 |
| 16 | 32 | 70 | 3.2 | 3.2 | 7.4 | 9 | 1.37903 | 0.8611 | 0.46517 | 0.39877 |
| 17 | 29 | 73 | 4.2 | 4.4 | 2 | 9 | 0.94381 | 1.07339 | 0.96437 | 1.21025 |
| 18 | 42 | 56 | 7.2 | 4.2 | 3.4 | 3.6 | 0.77687 | 1.14622 | 0.51538 | 0.39121 |
| 19 | 25 | 53 | 4.4 | 3.6 | 3 | 2.8 | 1.21991 | 0.65156 | 1.13355 | 1.1123 |
| 20 | 28 | 61 | 6.4 | 4.2 | 7.8 | 3.4 | 0.66487 | 0.95802 | 0.08991 | 1.06564 |
| 21 | 31 | 77 | 7.2 | 3.2 | 3.6 | 5 | 0.81872 | 1.009967 | 0.98989 | 1.0423 |
| 22 | 25 | 72 | 3.2 | 2.8 | 3 | 3.6 | 0.92256 | 0.64804 | 1.13031 | 1.02749 |
| 23 | 28 | 67 | 2.2 | 1.2 | 3 | 3.4 | 1.23801 | 1.11079 | 0.91633 | 1.0703 |
| 24 | 33 | 58 | 1.2 | 3.6 | 2.8 | 4 | 0.9045 | 0.69106 | 1.49063 | 0.84631 |
| 25 | 34 | 66 | 3.2 | 2.4 | 3 | 3 | 0.74899 | 1.06558 | 0.49146 | 0.85926 |
| 26 | 31 | 70 | 3.2 | 1.6 | 5.2 | 2.6 | 0.67709 | 0.48779 | 0.49146 | 0.11506 |
| 27 | 34 | 67 | 5.4 | 3.4 | 3.2 | 3.6 | 0.97446 | 0.13767 | 1.45277 | 0.8777 |
| 28 | 33 | 50 | 5 | 2.8 | 4.2 | 4.4 | 0.53796 | 1.03583 | 1.11253 | 1.15597 |

Control group

| No. | qRT-PCR  2^-ΔΔCt^ | | | |
| --- | --- | --- | --- | --- |
|  | Cx43 | | VEGF | |
|  | villi | decidua | villi | decidua |
| 1 | 0.3614061 | 5.796773 | 0.06675987 | 0.229514 |
| 2 | 2.470996 | 1.957008 | 0.9761164 | 0.219149 |
| 3 | 1.493185 | 3.728641 | 4.686221 | 0.56906 |
| 4 | 0.9191632 | 0.7838503 | 5.521748 | 11.20952 |
| 5 | 1.900913 | 0.1749786 | 0.5453011 | 0.103425 |
| 6 | 3.702226 | 6.169912 | 8.785503 | 4.397405 |
| 7 | 0.6933678 | 0.5195196 | 0.6728818 | 0.13428 |
| 8 | 1.190603 | 2.925433 | 0.9044577 | 3.145565 |
| 9 | 0.4327761 | 0.7659117 | 0.6822749 | 1.216992 |
| 10 | 0.9428452 | 0.5341253 | 0.0203592 | 1.697391 |
| 11 | 0.6636032 | 0.7347116 | 2.704056 | 6.133253 |
| 12 | 1.645348 | 1.291144 | 10.96721 | 1.940801 |
| 13 | 0.5658113 | 1.196359 | 12.33876 | 7.799163 |
| 14 | 0.9783298 | 0.4869853 | 0.1242937 | 0.749146 |
| 15 | 1.907513 | 0.9321603 | 1.296952 | 4.85673 |
| 16 | 1.03175 | 2.997272 | 26.94275 | 0.205421 |
| 17 | 1.390008 | 0.4810471 | 0.04581059 | 0.478848 |
| 18 | 1.003536 | 0.9024695 | 1.238355 | 0.106332 |
| 19 | 1.358197 | 1.435895 | 18.57287 | 2.584679 |
| 20 | 1.355094 | 0.4544678 | 1.600392 | 0.465104 |
| 21 | 1.342659 | 2.07333 | 1.890051 | 2.051462 |
| 22 | 0.4199801 | 0.46254 | 0.1344485 | 0.472456 |
| 23 | 1.672137 | 1.695781 | 2.068271 | 1.229711 |
| 24 | 0.5291296 | 0.7802184 | 2.805825 | 16.71787 |
| 25 | 0.08449987 | 0.3874145 | 0.02426676 | 0.571696 |
| 26 | 1.531657 | 0.5002253 | 0.762297 | 0.442522 |
| 27 | 1.160779 | 0.7467256 | 0.07939132 | 0.222723 |
| 28 | 1.503571 | 0.5593992 | 2.522973 | 1.647165 |

Case group

| No. | qRT-PCR  2^-ΔΔCt^ | | | |
| --- | --- | --- | --- | --- |
|  | Cx43 | | VEGF | |
|  | villi | decidua | villi | decidua |
| 1 | 0.3466839 | 0.1729767 | 0.7080019 | 0.3651731 |
| 2 | 0.3242308 | 0.3499571 | 0.7329695 | 1.245437 |
| 3 | 0.4258428 | 0.5329049 | 2.258124 | 1.200237 |
| 4 | 0.8695813 | 1.699664 | 0.009832848 | 0.04019953 |
| 5 | 0.5953017 | 0.4224786 | 0.2764566 | 0.5788395 |
| 6 | 0.3333461 | 0.3146784 | 0.2468703 | 0.1003644 |
| 7 | 1.234043 | 1.503839 | 2.252965 | 2.051462 |
| 8 | 0.2942456 | 0.3673558 | 0.0848951 | 0.05403291 |
| 9 | 0.6636032 | 1.247163 | 1.846925 | 0.2588136 |
| 10 | 1.581938 | 0.2038034 | 0.07811942 | 0.2942237 |
| 11 | 0.5254745 | 0.3403875 | 0.4606762 | 1.004622 |
| 12 | 0.5218449 | 0.4338445 | 0.05798506 | 0.8103698 |
| 13 | 0.5134846 | 0.447094 | 0.1977603 | 0.3904797 |
| 14 | 0.7855053 | 2.40936 | 2.482554 | 0.2111957 |
| 15 | 0.2367921 | 0.8077328 | 1.352028 | 0.9416869 |
| 16 | 0.1866481 | 0.5053133 | 0.02899253 | 0.06149668 |
| 17 | 0.1437559 | 0.5065055 | 0.08372632 | 0.2354217 |
| 18 | 0.524274 | 0.420055 | 0.3973342 | 0.04429606 |
| 19 | 0.4308008 | 0.3955549 | 1.731263 | 3.555335 |
| 20 | 0.6659071 | 1.221497 | 3.204557 | 0.9438649 |
| 21 | 0.3564305 | 0.1566102 | 0.0320935 | 0.06315244 |
| 22 | 3.74533 | 1.073223 | 6.97322 | 0.3824437 |
| 23 | 0.5315925 | 0.4926551 | 0.144769 | 0.5651291 |
| 24 | 0.3387735 | 0.5255682 | 0.03738038 | 4.438233 |
| 25 | 0.4180342 | 1.336676 | 3.868636 | 7.926326 |
| 26 | 0.5939416 | 0.733033 | 0.06463346 | 0.1406309 |
| 27 | 0.3715666 | 0.4747533 | 0.05920345 | 0.2606138 |
| 28 | 0.7047057 | 0.1225931 | 0.1516088 | 0.3238324 |
